# Comparison of Skeletal, Dental, and Soft Tissue Changes Before and After Orthodontic Treatment in Patients with Congenitally Missing Bilateral Maxillary Lateral Incisors

**DOI:** 10.3390/medicina61030485

**Published:** 2025-03-11

**Authors:** Tuğba Şenel, Orhan Cicek

**Affiliations:** Department of Orthodontics, Faculty of Dentistry, Zonguldak Bulent Ecevit University, Zonguldak 67600, Türkiye; tugba.senel@beun.edu.tr

**Keywords:** congenitally missing bilateral maxillary lateral incisors, space opening, space closure, orthodontic treatment, lateral cephalometry

## Abstract

(1) *Background and Objectives*: Congenitally missing bilateral maxillary lateral incisors (CMBMLIs) present significant aesthetic, functional, and psychosocial challenges that require an orthodontic approach based on multidisciplinary consensus. The aim of this study was to evaluate the skeletal, dental, and soft tissue changes in patients with CMBMLIs treated with space opening and closure methods and to compare these changes with those in untreated individuals. (2) *Materials and Methods*: A total of 53 patients (mean age 16 ± 3.5 years) were included, and three groups were formed: the study groups, consisting of the space opening group (*n* = 18) and the space closure group (*n* = 17), and the control group (*n* = 18), which had ideal occlusion. A total of 14 angular and 13 linear measurements were performed on lateral cephalograms before (T0) and after (T1) treatment. Statistical significance was set at *p* < 0.05. (3) *Results*: Compared to the control group, significant post-treatment changes were more evident in dental measurements and less evident in skeletal and soft tissue measurements. A statistically significant increase in the U1/SN angle was observed in the space opening group compared to the space closure group. The U1/NA angle increased significantly in both study groups, with a greater increase in the space opening group. However, although the change in U1/NA angle was not significantly different between groups, the increase was greater in the space opening group. No significant differences were found between the control and study groups in the nasolabial angle, upper lip length and thickness, and the distance from the upper and lower lips to the E-line. (4) *Conclusions*: While space opening and closure methods had minimal effects on most skeletal and soft tissue parameters, the space opening method significantly altered the maxillary incisor position. Considering the waiting period for prosthetic restoration after space opening and potential alveolar bone limitations, space closure is recommended for CMBMLIs when feasible because it ensures a more predictable planned maxillary incisor position.

## 1. Introduction

Dental anomalies may have a detrimental effect on aesthetics, chewing function, and speech, while also contributing to psychosocial challenges by affecting self-esteem and social interactions. Congenital tooth agenesis is a common dental anomaly characterized by the absence of one or more teeth, resulting in a total number of teeth in the mouth that is less than typical [1]. The prevalence of congenitally missing teeth varies across communities, continents, and genders, and the number of missing teeth also varies significantly based on these factors [2]. Some authors have suggested that the maxillary lateral incisors are the most common missing teeth in the permanent dentition, after the third molars [3,4].

The etiology of congenital dental agenesis is not fully understood, and various studies suggest that genetic, environmental, physical, chemical, and biological factors may contribute to the development of this condition [1]. Numerous studies have reported that congenital tooth missing is influenced by genetic factors, particularly mutations in the MSX1 and PAX9 genes, as well as by environmental factors, such as endocrine gland diseases, trauma to the head and neck region, developmental anomalies, and medical treatments [1,5]. Congenital missing teeth are not only a dental condition but also an indicator of diseases and syndromes, such as cleft lip and palate, Down syndrome, and ectodermal dysplasia, which develop from the ectodermal structure [6].

The effect of congenital tooth agenesis on craniofacial features and soft tissue profile remains a controversial issue in the literature. In addition to researchers who have found that individuals with congenital missing teeth have differences in craniofacial characteristics, there are also studies that have found that these individuals either do not differ significantly from the craniofacial characteristics of the unaffected population or have minimal differences [7]. Transversely, it has been reported that the maxillary intercanine width and the intercanine alveolar and skeletal widths are significantly reduced in congenital maxillary incisor agenesis [8]. On the other hand, a significant tendency toward skeletal Class III was observed sagittally in congenital maxillary incisor agenesis, attributed to maxillary hypoplasia and retrognathia [8,9].

The treatment of congenital tooth agenesis requires a comprehensive diagnostic evaluation and a multidisciplinary approach involving the collaboration of various dental specialists. In multidisciplinary treatment planning, the primary goals are to preserve existing teeth, achieve optimal esthetics, facilitate comfortable nutritional function, improve speech, reduce psychological distress associated with tooth loss, and ensure social acceptance [10]. It has been reported that a thorough assessment of the patient’s dentoalveolar developmental status, growth direction, and overall profile is essential when developing a treatment plan [11].

Due to the anterior location of the malocclusion, patients with congenitally missing bilateral maxillary lateral incisors (CMBMLIs) often seek orthodontic treatment with high aesthetic expectations [12]. Treatment options include closure methods, where the canines are moved to the lateral incisor position and the first premolars are repositioned to the canine position, or space opening methods, which are used for prosthetic rehabilitation to create adequate space for the placement of prosthetic restorations [13,14]. However, the literature remains controversial as to which method is superior [12]. On the other hand, it has been reported that the reduced buccolingual alveolar bone width after space opening treatment for CMBMLIs should be supported by bone grafting, or dental implants should be located palatally [15]. In contrast, with space closure, as the midfacial deficiency increases, a more concave profile becomes apparent and the canine crown will need to be polished and reshaped with composite to resemble a typical lateral incisor [12]. Therefore, given the potential limitations of both methods, careful diagnosis and an individualized treatment plan should be developed after a comprehensive interdisciplinary consultation [14]. Factors to consider, with an emphasis on choosing the least invasive treatment option whenever possible, include tooth position, occlusion, alveolar bone status, facial analysis, periodontal status, and the patient’s parafunctional habits [16].

Deteriorating smile aesthetics can lead to loss of self-confidence and social isolation. Particularly during childhood and adolescence, this condition can negatively affect self-perception and social interactions. Research indicates that both the aesthetic and functional consequences of tooth loss have a significant impact on an individual’s quality of life [17,18]. Particularly in cases such as CMBMLIs, the direct impact on facial aesthetics and dental function can prevent individuals from feeling comfortable in social settings. This condition can lead to psychosocial problems in adolescents, including a lack of self-confidence, difficulty communicating with peers, and even a decline in academic performance [19]. Therefore, accurate diagnosis and treatment of CMBMLIs not only enhances dental and functional rehabilitation, but also supports the psychosocial well-being of the individual. Appropriate intervention can enhance patient confidence, improve treatment compliance, and contribute to more successful long-term clinical outcomes [20].

To the best of our knowledge, no study has comprehensively evaluated the effects of orthodontic space opening and closure methods on dental, skeletal, and soft tissue changes in patients with CMBMLIs. In this regard, our study is expected to provide valuable clinical insights for orthodontists by comparing the results of space opening and space closure treatments in patients with CMBMLIs to each other and to individuals with ideal occlusion.

Therefore, the aim of this study was to evaluate the skeletal, dental, and soft tissue changes resulting from orthodontic treatment using space opening and space closure methods in patients with CMBMLIs by analyzing lateral cephalometric radiographs taken before (T0) and after treatment (T1) and comparing the results with those of individuals with ideal occlusion. The hypothesis of this study (H1) is that the methods of space opening and closure applied to patients with CMBMLIs cause significant differences in dental, skeletal, and soft tissue parameters compared to the control group.

## 2. Materials and Methods

### 2.1. Study Design, Sample Size Calculation, and Criteria

This retrospective, single-center study was conducted on the before- and after-treatment lateral cephalometric radiographs of patients with CMBMLIs and a skeletal Class I relationship, who presented to the Department of Orthodontics, Faculty of Dentistry, Zonguldak Bülent Ecevit University, Turkiye. The sample size for this study was calculated using the G*Power program (version 3.1.9.7; Franz Faul, University of Kiel, Kiel, Germany) with an U1/NA angle. Accordingly, the effect size of 0.5314202 obtained from the mean and standard deviations, with a 5% α error probability and 80% power (1-β err prob), indicated that a minimum of 39 total samples (13 per group) were required, and the actual power of this study was calculated to be 0.8203212 (critical F: 3.2594463 and non-centrality parameter λ: 11.0138897). A total of 53 patients were included in this study, and the inclusion criteria for the study groups (space opening, *n* = 18; space closure, *n* = 17) and the control group (*n* = 18) are shown in Table 1. The patients in the study and control groups who did not meet at least one of the inclusion criteria and those with unilateral congenitally missing maxillary lateral incisors were excluded from this study.

### 2.2. Ethical Approval and Consent

This study was approved by Zonguldak Bülent Ecevit University Non-Interventional Clinical Research Ethics Committee (date: 6 April 2022 and protocol no: 2022/07-2). Due to its retrospective design, this study was carried out in its first step by scanning lateral cephalometric radiographs from the archive of the Department of Orthodontics Clinic. All patients were informed before treatment began that their orthodontic records may be used for research purposes. In addition, informed consent was obtained from all patients and their legal guardians before treatment began. Due to the retrospective nature of this study, additional consent was not required. The gender, age, and treatment duration distribution of the patients enrolled in this study is shown in Table 2.

### 2.3. Orthodontic Interventions and Cephalometric Measurements

The patients in the study groups underwent fixed orthodontic treatment with 0.022 × 0.028-inch MBT^TM^ stainless steel metal brackets (Mini Master Series, American Orthodontics, Sheboygan, WI, USA), using the MBT^TM^ prescription. The treatment included the application of 0.012-inch, 0.014-inch, and 0.016-inch heat-activated NiTi round wires, followed by 0.016-inch stainless steel round wire, 0.019 × 0.025-inch heat-activated NiTi square wire, and 0.019 × 0.025-inch stainless steel rectangular wires (American Orthodontics, Sheboygan, WI, USA) [25]. In the space opening group, space opening mechanics, using NiTi open-coil springs, were applied with a 0.019 × 0.025-inch rectangular stainless steel wire. In the space closure group, one session after the placement of a 0.019 × 0.025-inch rectangular stainless steel wire (Brass posted wire, American Orthodontics, Sheboygan, WI, USA), a 1.5 mm × 8 mm temporary anchorage device (Aarhus System, American Orthodontics, Sheboygan, WI, USA) was placed in the alveolar bone, between the roots of the maxillary central incisors, at the level of the root midline, under local anesthesia. After providing indirect anchorage with the miniscrew, the spaces were closed using tiebacks with minimal anchorage [26].

**Figure 1:**
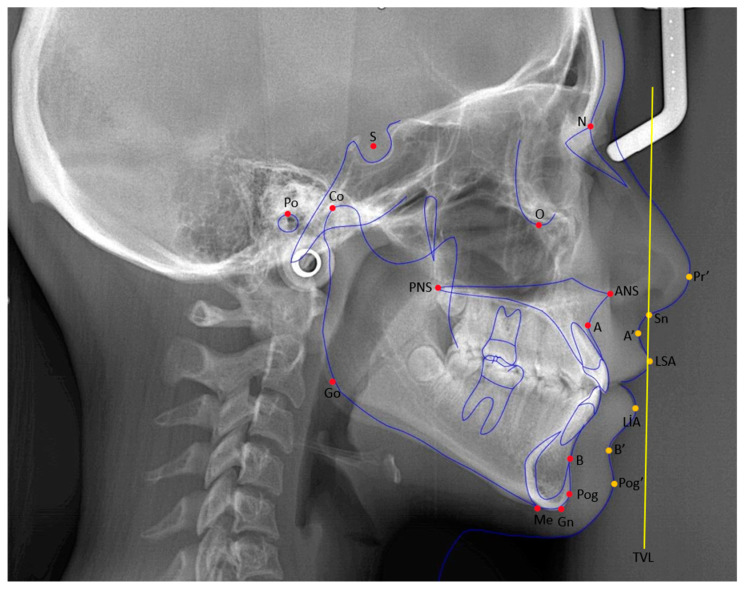
Red-colored points: cephalometric points related to hard tissue, yellow-colored points: cephalometric points related to soft tissue, blue-colored drawing: cephalometric illustration of hard and soft tissues with lines, yellow-colored vertical line: TVL—true vertical line.

The lateral cephalometric radiographs were traced and analyzed using the Nemoceph program (Nemotec, 2020, Madrid, Spain). After tracing the soft and hard tissue points on the cephalometric radiographs, 13 linear and 14 angular measurements were made separately at T0 and T1, using cephalometric analysis. All measurements were performed by the same researcher (TŞ). The definitions of the cephalometric points used in this study are shown in Table 3 [23,27]. The hard and soft tissue points used in this study are shown in Figure 1.

**Figure 2:**
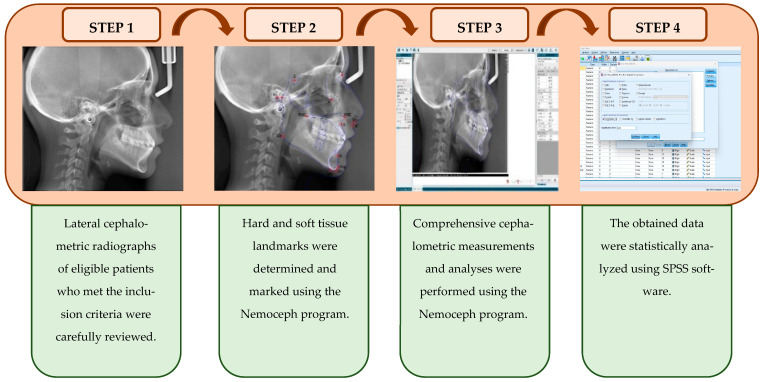
A graphical abstract of the methodological framework and stages of the study.

To provide a clear visualization of the study stages, the steps were applied sequentially and presented in detail through a graphical abstract. This visual abstract was designed to enhance comprehension of the data collection process and the methodological framework, illustrating how each step follows and interrelates with the previous one. Each step contributes to the overall purpose of this study, ensuring proper implementation of the data collection and methodological procedures. A graphical abstract of the steps of the data collection process and the methodological framework is presented in Figure 2.

The definitions of the other angular and linear measurements used in this study were specified and the corresponding measurement parameters were carefully recorded to increase the reliability of the data. These measurements are crucial for the accurate assessment of patients’ clinical conditions and cephalometric analyses. Therefore, the patients’ overjet and overbite distances, as well as their SNA, SNB, ANB, GoGn/SN, interincisal, and IMPA angles were recorded. In summary, these analyses of patient data provide a more complete understanding, including clear definitions for each angular and linear measurement. The definitions of the other angular and linear measurements used in this study are shown in Table 4 [23,27].

### 2.4. Statistical Analysis

The SPSS (Statistical Package for Social Sciences) 28.0 (IBM SPSS, Chicago, IL, USA) program was used for statistical analysis. The mean, standard deviation, and median values were used in the descriptive statistics of the data. The distribution of variables was measured by the Shapiro–Wilk test. ANOVA (Tukey test and Tamhane test) and an independent sample *t*-test were used to analyze normally distributed quantitative independent data, while Kruskal–Wallis and Mann–Whitney U tests were used for non-normally distributed data. Paired *t*-test and Wilcoxon tests were used to analyze normally and non-normally distributed dependent quantitative data, respectively. The statistical significance level was accepted as *p* < 0.05.

## 3. Results

### 3.1. Method Error

Reliability between the repeated linear and angular measurements was assessed using Cronbach’s α and two-way random effects intraclass correlation coefficients. The measurements of 18 patients, randomly selected by the same examiner (TŞ), were repeated on lateral cephalograms after four weeks. The reliabilities of the angular and linear measurements were found to be positive and strong, ranging from 0.89 to 0.99 (*p* < 0.001).

### 3.2. Results of Skeletal Measurements

The SNA angle in the control group was significantly (*p* < 0.05) higher than the space closure group at T0. There was no significant difference between the space opening group and the control and space closure groups in terms of SNA angle at T0 (*p* > 0.05). The SNA angle at T1 was significantly higher in the space opening than in the space closure (*p* < 0.05), but not in the control group (*p* > 0.05). The T0/T1 SNA change within and between groups did not show a significant difference (*p* > 0.05).

The SNB angle at T0 and T1 was significantly higher in the control and space opening group than in the space closure group (*p* < 0.05). There was no significant difference in SNB angle at T0 and T1 between the control group and the space opening group (*p* > 0.05). While the change in the SNB angle at T0/T1 was not significantly different in the space opening and space closure groups, the change in the space opening group was significantly higher than the space closure group (*p* < 0.05).

There was no significant difference in the ANB angle at T0, T1, or in the T0/T1 changes between the groups (*p* > 0.05).

There was no significant difference in the GoGn/SN angle at T0 between the control, space opening, and space closure groups (*p* > 0.05). The GoGn/SN angle at T1 was significantly higher in the space closure group than in the space opening group (*p* < 0.05). The GoGn/SN angle change within and between groups did not show a significant difference (*p* > 0.05).

The A Point–Nasion perpendicular distance at T0 and T1, as well as the T0/T1 changes in this value, did not show a significant difference between the groups (*p* > 0.05). The effective maxillary length value at T0 was significantly higher in the control group than in the space opening and space closure groups (*p* < 0.05). The effective maxillary length value at T0 was not significantly different between the space opening and space closure groups (*p* > 0.05). The effective maxillary length value at T1 in the space closure group was significantly lower than in the control group (*p* < 0.05). In the space opening and space closure group, the effective maxillary length value at T1 was significantly increased compared to T0 (*p* < 0.05). There was no significant difference in the T0/T1 changes in effective maxillary length between the space opening and space closure groups (*p* > 0.05).

The statistical analysis results of the skeletal measurements are shown in Table 5.

### 3.3. Results of Dental Measurements

The U1/SN angle and the U1/NA (mm) values at T0 were significantly higher in the control group than in the space opening and space closure groups (*p* < 0.05). There was no significant difference in the U1/SN angle and the U1/NA (mm) values at T0 between the space opening and space closure groups (*p* > 0.05). In the space opening group, the U1/SN angle value at T1 was significantly higher than the control and space closure group (*p* < 0.05), while the U1/NA (mm) value was significantly higher than the space closure group (*p* < 0.05). In the control and space closure groups, no significant difference was found in either parameter at T1 (*p* > 0.05). In the space opening group, the U1/SN angle and the U1/NA (mm) values increased significantly at T1 compared to T0 (*p* < 0.05), while no significant change was observed in the space closure group. The increase in the T0/T1 U1/SN angle in the space opening group was significantly higher than in the space closure group (*p* < 0.05). The increase in the T0/T1 U1/NA (mm) value in the space opening group was not significantly different from the space closure group (*p* > 0.05).

When the U1/NA angle was analyzed, it was observed that the values were significantly higher in the control group at T0 than in the space closure group (*p* < 0.05). The U1/NA angle at T0 was not significantly different between the space opening group and the control and space closure groups (*p* > 0.05). The U1/NA angle was not significantly different between the groups at T1 (*p* > 0.05). In the space opening and space closure groups, the U1/NA angle at T1 increased significantly compared to T0 (*p* < 0.05), whereas the T0/T1 changes between the groups were not significant (*p* > 0.05).

The U1-ANSPNS angle at T0 was significantly higher in the control group than in the space closure group (*p* < 0.05). There was no significant difference in the U1-ANSPNS angle at T0 between the space opening group and the control and space closure groups (*p* > 0.05). The U1-ANSPNS angle at T1 was significantly higher in the space opening group than in the space closure group (*p* < 0.05). There was no significant difference in the U1-ANSPNS angle at T1 between the control group and the space opening and space closure groups (*p* > 0.05). In the space opening group, the T1 U1-ANSPNS angle increased significantly compared to T0 (*p* < 0.05), while in the space closure group, the T1 U1-ANSPNS angle did not change significantly compared to T0 (*p* > 0.05). The change in the T0/T1 U1-ANSPNS angle between the space opening and space closure groups did not show a significant difference (*p* > 0.05).

The IMPA value at T0 was significantly higher in the control group than in the space closure group (*p* < 0.05), while the IMPA value at T0 was not significantly different between the space opening group and the control and space closure groups (*p* > 0.05). The IMPA value at T1 was significantly higher in the space opening group than in the space closure (*p* < 0.05). The IMPA value at T1 was not significantly different between the space opening and space closure groups compared to the control (*p* > 0.05). The T0/T1 IMPA change within and between groups did not differ significantly (*p* > 0.05).

The interinsisal angle at T0 was significantly higher in the space closure group than in the control group (*p* < 0.05). The interinsisal angle at T0 was not significantly different between the space opening group and the control and space closure groups (*p* > 0.05). The interinsisal angle at T1 was not significantly different between the control, space opening, and space closure groups (*p* > 0.05). In the space opening, the interinsisal angle at T1 showed a significant decrease compared to T0 (*p* < 0.05), while in the space closure group, the interinsisal angle at T1 did not show a significant change compared to T0 (*p* > 0.05). The T0/T1 interinsisal angle change did not differ significantly between the space opening and space closure groups (*p* > 0.05).

The U1–Upper occlusal line value at T0 was significantly higher in the space closure group than in the control group (*p* < 0.05). The U1–Upper occlusal line value at T0 was not significantly different between the space opening and the control and space closure groups (*p* > 0.05). The U1–Upper occlusal line value at T1 was significantly higher in the space closure group than in the control and space opening groups (*p* < 0.05). The U1–upper occlusal line value at T1 was not significantly different between the control and space opening groups (*p* > 0.05). The U1–upper occlusal line value at T1 in the space opening group showed a significant decrease compared to T0 (*p* < 0.05), while the U1–upper occlusal line value at T1 in the space closure group showed no significant change compared to T0 (*p* > 0.05). No significant difference was observed in the U1–upper occlusal line value at T0/T1 between the groups (*p* > 0.05).

The L1/NB angle and the L1/NB (mm) value at T0 and T1 did not differ significantly between the groups (*p* > 0.05). In the space opening group, the L1/NB angle did not change significantly at T1 compared to T0 (*p* > 0.05), while the L1/NB (mm) value increased significantly (*p* < 0.05). In the space closure group, the L1/NB angle and the L1/NB (mm) value did not change significantly at T1 (*p* > 0.05). The L1/NB angle and the L1/NB (mm) value at T0/T1 were not significantly different between groups (*p* > 0.05).

The upper incisor incisal–TWL value at T0 and T1 was not significantly different between the groups (*p* > 0.05). In the space opening group, the upper incisor incisal–TWL value at T1 showed a significant decrease compared to T0, while in the space closure group, the upper incisor incisal–TWL value at T1 showed a significant increase compared to T0 (*p* < 0.05). The T0/T1 upper incisor incisal–TWL change between the groups showed a significant difference (*p* < 0.05).

The overjet value at T0 was significantly higher in the control and space closure group than in the space opening group (*p* < 0.05). The overjet value at T0 was not significantly different between the control and space closure group (*p* > 0.05). While the overjet value at T1 was significantly higher in the space opening group than in the space closure group (*p* < 0.05), the overjet value at T1 was not significantly different between the control group and the space opening and space closure groups (*p* > 0.05). The overjet value at T1 in the space opening group showed a significant increase compared to T0 (*p* < 0.05), while no significant change was observed in the space closure group (*p* > 0.05). The T0/T1 overjet increase in the space opening group was significantly higher than the space closure group (*p* < 0.05). The overbite values at T0, T1, and the T0/T1 change were not significantly different within and between groups (*p* > 0.05).

The statistical results of the angular and linear dental cephalometric measurements are presented in Table 6 and Table 7, respectively.

### 3.4. Results of Soft Tissue Measurements

There was no significant difference in the nasolabial angle value at T0, T1, and the T0/T1 change between the control, space opening, and space closure groups (*p* > 0.05).

The labiomental angle value at T0 was significantly higher in the space closure group than in the control group (*p* < 0.05). While the labiomental angle value at T1 in the space closure group was significantly higher than the control and space opening groups (*p* < 0.05), the labiomental angle value at T1 did not differ significantly between the control group and the space opening group (*p* > 0.05). The T0/T1 change within and between groups did not show a significant difference (*p* > 0.05).

There was no significant difference in upper lip length and upper lip thickness at T0, T1, and the T0/T1 change between the control, space opening, and space closure groups (*p* > 0.05). The upper lip angle at T0 was not significantly different between the groups (*p* > 0.05). While the upper lip angle at T1 was significantly higher in the space opening group than in the space closure group (*p* < 0.05), the upper lip angle at T1 was not significantly different between the control group and the space opening and space closure groups (*p* > 0.05). In the space opening group, there was a significant increase in the upper lip angle at T1 compared to T0 (*p* < 0.05), while in the space closure group, there was a significant decrease in the upper lip angle at T1 compared to T0 (*p* < 0.05). The T0/T1 upper lip angle change between space opening and space closure groups was found to be significant (*p* < 0.05).

There was no significant difference in the upper lip–E line and lower lip–E line values at T0, T1, and the T0/T1 change within and between the groups (*p* > 0.05). The A’-TVL value at T0 was significantly lower in the space closure group than in the control and space opening group (*p* < 0.05). The A’-TVL value at T0 was not significantly different between the control and space opening group (*p* > 0.05). The T1 A’-TVL value did not differ significantly between the groups (*p* > 0.05). In the space opening group, the A’-TVL value at T1 did not change significantly compared to T0 (*p* > 0.05), whereas in the space closure group, the A’-TVL value at T1 increased significantly compared to T0 (*p* < 0.05). In the space opening group, the T0/T1 A’-TVL change was significantly lower than in the space closure group (*p* < 0.05).

The upper lip anterior portion–TVL value did not differ significantly between the groups at T0 and T1 (*p* > 0.05). In the space opening and space closure groups, the upper lip anterior portion–TVL value at T1 did not show a significant change compared to T0 (*p* > 0.05). In the space opening group, the T0/T1 change in the upper lip anterior part–TVL distance was significantly lower than in the space closure group (*p* < 0.05).

The statistical results of the soft tissue measurements are shown in Table 8.

## 4. Discussion

Due to their position along the smile line and their visibility during speech, the congenital missing of maxillary lateral incisors is more noticeable and often leads patients to seek treatment at orthodontic clinics for aesthetic reasons [28]. There are studies in the literature that have examined changes in the craniofacial structures in patients with congenital missing teeth [8,29,30]. However, no study has comprehensively examined how different treatment approaches for congenital CMBMLIs affect skeletal, dental, and soft tissue. The aim of this study was to comprehensively analyze skeletal, dental, and soft tissue changes using lateral cephalometric radiographs taken before and after orthodontic treatment in patients with interdental spaces due to congenital CMBMLIs, thereby improving the accuracy of orthodontic treatment planning. The criteria for selecting reference points, as well as linear and angular measurements in this study, were their ease of reproducibility, ability to reflect before- and after-treatment changes, and suitability for comparison with previous studies. The parameters utilized in this study are consistent with those used in previous research, which ensures comparability and strengthens the reliability of the results [31,32,33].

The effect of congenital maxillary missing teeth on skeletal parameters remains a topic of ongoing debate and controversy in the literature, with conflicting results reported in different studies. Numerous studies have shown that congenitally missing maxillary teeth adversely affect maxillary development, often resulting in retropositioned maxilla and associated craniofacial changes, with potential functional and aesthetic implications [32,34]. In another study conducted on patients with congenital CMBMLIs, it was reported that a significant difference was observed only in the SNA angle when compared to the control group in terms of skeletal measurements, while no significant differences were found in other skeletal parameters [31]. In this study, we observed that treatment with both the space opening and space closure methods did not result in significant differences in skeletal parameters, including the SNA, SNB, ANB, GoGn/SN, A point ⊥ Nasion Perpendicular, and effective maxillary length. We believe that the absence of significant changes in skeletal angles may be attributed to the limited effect of space opening and space closure treatments on hard tissues. In addition, the observed changes in effective maxillary length are likely to be the result of tooth movement affecting the position of point A, along with the ongoing growth and development of individuals, to some extent.

Endo et al. [35] reported that in patients with congenital missing teeth, the maxillary and mandibular incisors were positioned more retrusively compared to the control group, likely due to changes in the balance of tongue–lip pressure and the lingual tilting of the lower incisors, which serves as a compensatory mechanism for the deficiencies in the anterior region. Another study reported that patients with congenital lateral incisors missing showed a statistically significant difference compared to the control group in the U1/NA°, L1/NB°, U1/NA (mm), and L1/NB (mm) measurements, while no significant difference was found in the U1/SN° angle [33]. In contrast, another study reported that there was no significant difference in the U1/NA angle and the L1/NB angle in patients with congenital CMBMLIs compared with the control group, but there was a statistically significant increase in the U1/NA (mm) and L1/NB (mm) values [36]. Another study from the literature found that the interincisal angle was significantly increased in individuals with CMBMLIs, which was attributed to a decrease in the inclination of the maxillary and mandibular incisor axes [31]. On the contrary, another study reported that there was no significant difference in the interincisal angle between the CMBMLI group and the control group [33].

Moreover, these angular changes are not only crucial for aesthetics and tooth alignment but also have significant functional implications. The inclination and position of the anterior incisors play a key role in lip support, speech function, and occlusal stability [37]. Specifically, alterations in the interincisal angle can affect lip closure dynamics, potentially modifying the patient’s habitual mouth closure position and muscular adaptation [23]. Additionally, the vestibulo–lingual positioning of the incisors directly influences the contact relationship between the maxillary and mandibular anterior teeth, thereby impacting occlusal balance [38]. In this context, evaluating the effects of space opening procedures on the incisor inclination is essential for ensuring both short- and long-term stability in orthodontic treatment.

In the present study, while significant changes were observed in the U1/NA (°) angle with both treatment methods, more pronounced and significant alterations were found in the U1/SN (°), U1/NA (mm), U1-ANSPNS (°), U1–Upper occlusal line (°), and interincisal angle, specifically, in the space opening group. It has been emphasized that these significant angular and linear changes should be carefully considered when planning the position of the incisors in orthodontic treatment, especially in patients undergoing space opening procedures, as these changes can have a profound effect on the overall treatment outcome and the alignment of the anterior teeth [39]. There is no consensus in the literature regarding soft tissue changes in patients with CMBMLIs, and this issue remains a topic of ongoing debate. Therefore, our study provides valuable insights for clinicians, offering a better understanding of soft tissue profile changes in patients with CMBMLIs and helping to inform treatment planning. These findings may contribute to more informed and effective treatment processes.

Bassiony et al. [8] conducted a study on patients with CMBMLIs and reported a significant increase in the upper lip–E line and lower lip–E line distances when compared to the control group. Additionally, they found that both the maxillary and mandibular lips were positioned more retrusively in the patient group. Motairi et al. [36] conducted a study on patients with CMBMLIs and found no significant difference in the lower lip–E line distance when compared to the control group. However, they observed a significant increase in the upper lip–E line distance, with the upper lip positioned more retrusively relative to the unaffected population. In addition, they reported that the nasolabial angle was significantly higher in the group with congenital missing teeth compared to the control group. In our study, we observed no significant difference in the nasolabial angle, upper lip–E line distance, and lower lip–E line distance in within-group and between-group comparisons at T0, T1, and the T0/T1 changes.

Öztürk et al. [33] conducted a study comparing patients with CMBMLIs to a control group and reported that upper lip thickness was significantly greater in individuals with a Class II skeletal relationship compared to those with a Class I skeletal relationship. Another study reported that lips became more retrusive as the severity of hypodontia increased, but not to a level that would cause a statistically significant difference [40]. In the present study, no significant differences in upper lip length and upper lip thickness were observed both within and between groups, regardless of the treatment method. While the upper lip angle did not differ significantly between groups at T0, it was significantly greater in the space opening group compared to the space closure group at T1. A significant increase in the upper lip angle was observed at T1 in the space opening group compared to T0, whereas the space closure group showed a significant decrease. The change in upper lip angle from T0/T1 was significantly less in the space closure group compared to the space opening group. The A’-TVL distance increased in the space opening group and decreased significantly in the space closure group.

Based on the results of our study, the hypothesis was more strongly supported by the dental measurements, especially those of the maxillary incisors. However, no significant differences were found in most results within or between groups for measures of both skeletal and soft tissue parameters, and therefore the hypothesis was rejected. These results should be carefully considered when treating CMBMLI patients with the space opening and space closure methods. It has become clear that the proclination of the maxillary incisors, especially in the space opening method, should not be expected to provide compensatory soft tissue effects in individuals with CMBMLIs with a Class III skeletal tendency and a concave profile.

It has been reported in the literature that the treatment of CMBMLIs with the space closing method is 87.5% more frequent than with the space opening method, and that suitable cases for the space opening method are those with Class I molar relationships, concave facial profiles, Class III malocclusions, and cases in which canine remodeling is not recommended [14,39,41]. On the other hand, the space closure method offers the advantage of completing the orthodontic treatment during the adolescent period [11,42], while eliminating the additional waiting time for prosthetic preparation and additional implant surgery [14]. In addition, it provides the growing child with an occlusion that naturally adapts to the normal craniofacial changes in growth [42,43].

The sample of our study, consisting of patients in treatment during adolescence, makes the clinical integration of the results more tangible and applicable. In this context, although no significant differences were found between the study and control groups at the skeletal and soft tissue levels, significant differences were observed in the measured parameters at the dental level, particularly those related to the maxillary incisors. The treatments performed during this period may take advantage of skeletal growth potential, thereby aiding in the preservation of maxillary arch length and the establishment of optimal anterior tooth relationships [42,43]. In addition, improved periodontal and alveolar tissue adaptation may facilitate a more physiologic completion of the space closure method. Consistent with the literature, it has been emphasized that treating CMBMLI patients with the space closure method during adolescence offers several advantages. These advantages include a shorter treatment duration compared to adults, better preservation of periodontal health, and greater long-term stability [44]. Moreover, addressing esthetic and functional concerns earlier in adolescence is a critical factor in promoting psychosocial well-being. Early intervention in this age group may increase patient confidence and positively influence treatment adherence [17]. Therefore, the results of this study provide important clinical evidence to support the preference of the space closure method in the treatment of adolescent CMBMLI patients.

The present study used lateral cephalometric radiography, which allows for the evaluation of angular and linear measurements. The reduction of three-dimensional structures to two dimensions for analysis is a limitation of this study. However, previous studies have shown that there is no statistically significant difference between lateral cephalometric radiographs and CBCT images, which enable three-dimensional assessment [45,46]. Other limitations of this study include the variability of spaces among the included patients and the unique soft tissue patterns in each individual. It is recommended that future studies address these limitations with a larger sample size. In addition, to the best of our knowledge, this study is the first to evaluate the pre- and post-treatment changes in orthodontic space opening and closure methods in patients with CMBMLIs by comparing them to individuals with ideal occlusion. Therefore, the results of this study are expected to provide clinical evidence to assist orthodontists in more precise and accurate treatment planning for CMBMLI patients.

## 5. Conclusions

Our results showed that both treatment methods had a lesser effect on soft tissue and skeletal parameters, but a greater effect on dental measurements. It was emphasized that when treating CMBMLI individuals, especially those with Class III tendencies and a concave profile, the maxillary central incisor should be carefully evaluated and its position pre-planned when using the space opening method. The maxillary incisor position should be determined based on the existing skeletal and soft tissue profiles, and the space closure method is preferred whenever possible because it:

(i) Ensures the planned maxillary incisor position,
(ii) Eliminates the waiting period for prosthetic restoration,
(iii) Provides a cost-effective solution,
(iv) Avoids potential anatomical disadvantages in the alveolar bone for future implant surgery.

## Figures and Tables

**Table 1:** The inclusion criteria for the study and control groups.

Study Groups (Space Opening and Closure)	Control Group
Having congenitally missing bilateral maxillary lateral incisors.	No missing teeth, except for the third molars.
No congenital tooth missing other than maxillary lateral incisors and third molars.	Having skeletal Class I relationship.
Having skeletal Class I relationship.	No or very mild (<2 mm) crowding/diastema.
No previous orthodontic, orthognathic, or prosthodontic treatment.	No previous orthodontic treatment history.
Having completed fixed orthodontic treatment.	Having normal overbite and overjet.
In the space opening group, the root position of the maxillary canine should be close to its original position (with at least 6–6.5 mm of intercoronal space and at least 5.7 mm of interradicular space opened) [21,22].	Having a smile with a gingival appearance of a maximum of 4 mm or lip–tooth coverage of a maximum of 2 mm [23].
In the space closure group, the maxillary canine should be close to the midline to a degree that it can be positioned in place of the lateral incisor (with the space being of mild severity) [24].	Having high-quality lateral cephalometric radiographs.
Having high-quality lateral cephalometric radiographs in the archive before (T0) and after (T1) orthodontic treatment.	

**Table 2:** Gender, age, and treatment duration distribution of the patients.

		Control	Space Opening	Space Closure
Age (year)	Mean ± sd	18.1 ± 4.1	15.4 ± 2.9	14.9 ± 2.9
Gender	Female (*n*-%)	15–83.3%	14–77.8%	14–82.4%
	Male (*n*-%)	3–16.7%	4–22.2%	3–17.6%
Treatment duration (year)			2.66 ± 1.07	3.41 ± 1.26

sd: standard deviation, *n*: sample.

**Table 3:** The important cephalometric points and their definitions used in this study.

Cephalometric Points	Definitions
Sella (S)	The geometric center of the sella turcica.
Nasion (N)	The most anterior and deepest point of the frontonasal suture.
A point (A)	The deepest point of the bony concavity on the anterior surface of the maxilla.
B point (B)	The deepest point of the bony concavity on the anterior surface of the mandible.
Porion (Po)	The uppermost point of the bony concavity that forms the external auditory canal.
Pogonion (Pog)	The most anterior point of the mandible in the sagittal plane.
Gnathion (Gn)	The most anterior and inferior point of the mandible.
Menton (Me)	The point where the lower border of the mandible meets the symphysis in the sagittal plane.
Gonion (Go)	The point where the bisector of the angle formed by the intersection of lines, drawn along the ramus and corpus of the mandible, cuts the ramus.
Maxillary incisor edge point	The point of the incisal edge of the most anteriorly positioned maxillary incisor.
Mandibular incisor edge point	The point of the incisal edge of the most anteriorly positioned mandibular incisor.
Subnasale (Sn)	The point of junction between the lower border of the nasal septum and the upper lip.
Labialis superior anterior (LSA)	The most anterior point of the upper lip in the sagittal plane.
Labialis inferior anterior (LIA)	The most anterior point of the lower lip in the sagittal plane.
Condylion (Co)	The most superoposterior point of the condyle in the sagittal plane.

**Table 4:** The angular and linear measurements used in this study.

	Parameters	Definitions
Angular measurements (°)	U1/SN	It is the angle that the axis inclination of the upper incisor makes with the SN plane.
	U1/NA	The angle between the inclination of the upper incisor axis and the NA line.
	L1/NB	It is the angle between the lower incisor axis inclination and the NB line.
	Nasolabial	It is the angle formed by the intersection of the most anterior point of the upper lip and the tangent passing through the columella in the subnasale region.
	Labiomental	It is the angle formed at the intersection of the most anterior point of the lower lip, the supramentale, and the lines drawn from the soft tissue pogonion to the supramentale.
	Upper Lip	It is the angle formed between the line drawn from the most anterior point of the upper lip to the subnasale point and the GDS.
	U1-ANSPNS	The angle formed at the intersection of the upper incisal axis inclination and the spinalar plane.
	U1–Upper Occlusal Plane	The angle formed between the inclination of the upper incisor axis and the upper occlusal plane.
Linear Measurements (mm)	U1/NA	The distance measured from the incisal of the upper incisor down to the NA line.
	L1/NB	The distance measured from the incisal of the lower incisor to the NB line.
	A Nasion Perpendicular (N⊥A)	It is the distance between the perpendiculars, lowered from the points Nasion and A to the Frankfurt horizontal plane.
	Effective Maxillary Length	This is the distance between the Condylon point and point A.
	Upper Lip Length (Sn-ULI)	The distance between the subnasale point and the lowest point of the upper lip.
	Upper Lip Thickness	The distance between the most anterior part of the upper lip and the inner part.
	Upper Lip–E Line	The distance between the most anterior point of the upper lip and the E plane.
	Lower Lip–E Line	The distance between the most anterior point of the lower lip and the E plane.
	A’-TVL (mm)	The distance between the soft tissue point A and the true vertical line.
	Upper Incisor Incisal—TVL	The distance between the incisal point of the upper incisor and the true vertical line.
	Upper Lip Anterior Part—TVL	It is the distance between the most anterior point of the upper lip and the true vertical line.

(°): degree, (mm): millimeter.

**Table 5:** Statistical results of skeletal measurements.

		Control ^1^	Space Opening ^2^	Space Closure ^3^	*p*
**SNA**					
T0	Mean ± sd	81.4 ± 1.4	81.6 ± 4.1	79.2 ± 2.8 ^1^	0.036 *^,K^
T1	Mean ± sd	81.4 ± 1.4	81.8 ± 4.3	78.6 ± 2.9 ^1,2^	0.006 *^,K^
T0/T1 Change	Mean ± sd		0.2 ± 1.2	−0.6 ± 1.3	0.077 ^M^
*Intra Group Change p*			0.415 ^W^	0.093 ^W^	
**SNB**					
T0	Mean ± sd	79.2 ± 1.1	79.9 ± 3.4	77.3 ± 3.0 ^1,2^	0.012 *^,K^
T1	Mean ± sd	79.2 ± 1.1	80.5 ± 3.7	76.8 ± 3.1 ^1,2^	0.002 *^,K^
T0/T1 Change	Mean ± sd		0.6 ± 1.3	−0.5 ± 1.3	0.025 *^,M^
*Intra Group Change p*			0.092 ^W^	0.103 ^W^	
**ANB**					
T0	Mean ± sd	2.3 ± 1.0	1.6 ± 1.4	1.9 ± 1.2	0.224 ^K^
T1	Mean ± sd	2.3 ± 1.0	1.4 ± 1.6	1.9 ± 1.5	0.183 ^K^
T0/T1 Change	Mean ± sd		−0.2 ± 0.6	−0.1 ± 0.6	0.397 ^M^
*Intra Group Change p*			0.157 ^W^	0.655 ^W^	
**GOGn/SN**					
T0	Mean ± sd	33.7 ± 3.0	32.4 ± 4.2	35.3 ± 4.9	0.129 ^K^
T1	Mean ± sd	33.7 ± 3.0	32.3 ± 4.6	35.9 ± 5.1 ^2^	0.045 *^,K^
T0/T1 Change	Mean ± sd		−0.1 ± 2.4	0.6 ± 2.1	0.242 ^M^
*Intra Group Change p*			0.905 ^W^	0.187 ^W^	
**A-Nazion Perpendicular**					
T0	Mean ± sd	−1.4 ± 2.5	−1.5 ± 4.2	−3.5 ± 2.8	0.115 ^A^
T1	Mean ± sd	−1.4 ± 2.5	−1.1 ± 4.8	−3.7 ± 3.5	0.101 ^A^
T0/T1 Change	Mean ± sd		0.4 ± 2.4	−0.2 ± 2.8	0.537 ^t^
*Intra Group Change p*			0.500 ^P^	0.817 ^P^	
**Effective Maxillar Length**					
T0	Mean ± sd	87.7 ± 4.2	83.3 ± 3.6^1^	81.4 ± 6.8 ^1^	0.001 *^,K^
T1	Mean ± sd	87.7 ± 4.2	87.1 ± 3.2	83.8 ± 6.1 ^1^	<0.050 *^,K^
T0/T1 Change	Mean ± sd		3.8 ± 3.4	2.4 ± 2.8	0.314 ^M^
*Intra Group Change p*			<0.001 *^,W^	<0.001 *^,W^	

^K^: Kruskal–Wallis test, ^M^: Mann–Whitney U test, ^A^: ANOVA test, ^t^: *t*-test, ^W^: Wilcoxon, ^P^: Paired *t*-test, T0: pre-treatment, T1: post-treatment, sd: standard deviation, *p*: significance level, *: *p* < 0.05, ^1^: difference with control group, ^2^: difference with space opening group, ^3^: difference with space closure group.

**Table 6:** Statistical results of angular (°) dental measurements.

		Control ^1^	Space Opening ^2^	Space Closure ^3^	*p*
**U1/SN**					
T0	Mean ± sd	104.3 ± 3.1	100.2 ± 4.5 ^1^	98.1 ± 7.5 ^1^	0.004 *^,A^
T1	Mean ± sd	104.3 + 3.1 ^2^	108.2 ± 3.2	100.0 ± 7.6 ^2^	<0.001 *^,A^
T0/T1 Change	Mean ± sd		8.0 ± 5.7	1.9 ± 6.0	0.004 *^,t^
*Intra Group Change p*			<0.001 *^,P^	0.198 ^P^	
**U1/NA**					
T0	Mean ± sd	24.1 ± 3.6	20.2 ± 4.6	19.9 ± 6.6 ^1^	0.031 *^,A^
T1	Mean ± sd	24.1 ± 3.6	27.2 ± 4.5	24.4 ± 5.7	0.099 ^A^
T0/T1 Change	Mean ± sd		7.0 ± 6.6	4.4 ± 7.9	0.300 ^t^
*Intra Group Change p*			<0.001 *^,P^	0.035 *^,P^	
**U1-ANSPNS**					
T0	Mean ± sd	113.7 ± 5.2	109.8 ± 5.5	107.8 ± 6.3^1^	0.012 *^,A^
T1	Mean ± sd	113.7 ± 5.2	116.6 ± 4.3	110.6 ± 6.3^2^	0.008 *^,A^
T0/T1 Change	Mean ± sd		6.8 ± 6.0	2.8 ± 6.6	0.070 ^t^
*Intra Group Change p*			<0.001 *^,P^	0.106 ^P^	
**IMPA**					
T0	Mean ± sd	91.5 ± 3.3	90.2 ± 5.5	86.4 ± 6.5 ^1^	0.016 *^,A^
T1	Mean ± sd	91.5 ± 3.3	92.4 ± 7.2	86.9 ± 7.2 ^2^	0.025 *^,A^
T0/T1 Change	Mean ± sd		2.3 ± 4.6	0.6 ± 7.4	0419 ^t^
*Intra Group Change p*			0.051 ^P^	0.746 ^P^	
**Interincisal Angle**					
T0	Mean ± sd	130.3 ± 6.5	136.9 ± 11.4	140.2 ± 9.6 ^1^	0.010 *^,A^
T1	Mean ± sd	130.3 ± 6.5	129.8 ± 9.0	134.9 ± 10.7	0.194 ^A^
T0/T1 Change	Mean ± sd		−7.1 ± 9.0	−5.3 ± 11.0	0.597 ^t^
*Intra Group Change p*			0.004 *^,P^	0.066 ^P^	
**U1–Upper Occlusal Plane**					
T0	Mean ± sd	57.6 ± 3.0	59.8 ± 4.8	62.1 ± 6.3 ^1^	0.016 *^,A^
T1	Mean ± sd	57.6 ± 3.0	55.2 ± 3.2	61.4 ± 5.7 ^1,2^	<0.001 *^,A^
T0/T1 Change	Mean ± sd		−4.6 ± 4.8	−0.6 ± 7.0	0.057 ^t^
*Intra Group Change p*			0.001 *^,P^	0.707 ^P^	
**U1-ANSPNS**					
T0	Mean ± sd	113.7 ± 5.2	109.8 ± 5.5	107.8 ± 6.3 ^1^	0.012 *^,A^
T1	Mean ± sd	113.7 ± 5.2	116.6 ± 4.3	110.6 ± 6.3 ^2^	0.008 *^,A^
T0/T1 Change	Mean ± sd		6.8 ± 6.0	2.8 ± 6.6	0.070 ^t^
*Intra Group Change p*			<0.001 *^,P^	0.106 ^P^	
**L1/NB**					
T0	Mean ± sd	23.2 ± 4.8	20.9 ± 8.5	17.9 ± 7.1	0.086 ^A^
T1	Mean ± sd	23.2 ± 4.8	21.7 ± 9.1	19.2 ± 7.9	0.283 ^A^
T0/T1 Change	Mean ± sd		0.8 ± 4.5	1.3 ± 7.3	0.801 ^t^
*Intra Group Change p*			0.471 ^P^	0.474 ^P^	

^A^: ANOVA test, ^t^: *t*-test, ^P^: Paired *t*-test, T0: pre-treatment, T1: post-treatment, sd: standard deviation, *p*: significance level, *: *p* < 0.05. ^1^: difference with control group, ^2^: difference with space opening group, ^3^: difference with space closure group.

**Table 7:** Statistical results of linear (mm) dental measurements.

		Control ^1^	Space Opening ^2^	Space Closure ^3^	*p*
**U1/NA**					
T0	Mean ± sd	5.2 ± 1.3	3.4 ± 1.9 ^1^	3.5 ± 2.1 ^1^	0.008 *^,K^
T1	Mean ± sd	5.2 ± 1.3	6.2 ± 1.8	4.5 ± 2.8 ^2^	0.008 *^,K^
T0/T1 Change	Mean ± sd		2.8 ± 2.7	1.0 ± 3.2	0.075 ^m^
*Intra Group Change p*			<0.001 *^,W^	0.379 ^W^	
**L1/NB**					
T0	Mean ± sd	4.8 ± 1.6	3.1 ± 3.2	3.4 ± 2.5	0.112 ^A^
T1	Mean ± sd	4.8 ± 1.6	4.0 ± 2.4	3.5 ± 2.6	0.260 ^A^
T0/T1 Change	Mean ± sd		0.9 ± 1.6	0.1 ± 2.1	0.207 ^t^
*Intra Group Change p*			0.028 *^,P^	0.876 ^P^	
**Upper Incisor-TVL**					
T0	Mean ± sd	12.7 ± 2.1	13.7 ± 2.3	11.8 ± 4.2	0.180 ^A^
T1	Mean ± sd	12.7 ± 2.1	12.2 ± 2.7	13.4 ± 3.6	0.483 ^A^
T0/T1 Change	Mean ± sd		−1.5 ± 2.2	1.6 ± 3.1	0.002 *^,t^
*Intra Group Change p*			0.011 *^,P^	<0.050 *^,P^	
**Overjet**					
T0	Mean ± sd	3.3 ± 0.4^2^	2.4 ± 0.8	3.2 ± 1.2 ^2^	0.001 *^,K^
T1	Mean ± sd	3.3 ± 0.4	3.8 ± 0.8	3.2 ± 0.9 ^2^	0.042 *^,K^
T0/T1 Change	Mean ± sd		1.5 ± 0.8	0.0 ± 1.7	<0.001 *^,M^
*Intra Group Change p*			<0.001 *^,W^	0.407 *^,W^	
**Overbite**					
T0	Mean ± sd	2.5 ± 0.9	2.7 ± 1.4	3.1 ± 1.4	0.348 ^K^
T1	Mean ± sd	2.5 ± 0.9	2.1 ± 0.9	2.4 ± 1.4	0.895 ^K^
T0/T1 Change	Mean ± sd		−0.6 ± 1.3	−0.6 ± 1.8	0.409 ^M^
*Intra Group Change p*			0.067 ^W^	0.109 ^W^	

^K^: Kruskal–Wallis test, ^M^: Mann–Whitney U test, ^A^: ANOVA test, ^t^: *t*-test, ^W^: Wilcoxon, ^P^: Paired *t*-test, T0: pre-treatment, T1: post-treatment, sd: standard deviation, *p*: significance level, *: *p* < 0.05. ^1^: difference with control group, ^2^: difference with space opening group, ^3^: difference with space closure group.

**Table 8:** Statistical results of soft tissue measurements.

		Control ^1^	Space Opening ^2^	Space Closure ^3^	*p*
**Nasolabial Angle**					
T0	Mean ± sd	109.5 ± 7.7	108.9 ± 7.6	109.8 ± 10.2	0.953 ^A^
T1	Mean ± sd	109.5 ± 7.7	105.0 ± 9.0	110.3 ± 10.3	0.180 ^A^
T0/T1 Change	Mean ± sd		−3.9 ± 9.7	0.5± 10.1	0.196 ^t^
*Intra Group Change p*			0.102 ^P^	0.850 ^P^	
**Labiomental Angle**					
T0	Mean ± sd	121.8 ± 10.3	125.2 ± 11.8	133.3 ± 16.5 ^1^	0.036 *^,A^
T1	Mean ± sd	121.8 ± 10.3	120.8± 12.6	132.6 ± 11.7 ^1,2^	0.007 *^,A^
T0/T1 Change	Mean ± sd		−4.4 ± 9.3	−0.6 ± 13.4	0.341 ^t^
*Intra Group Change p*			0.062 ^P^	0.844 ^P^	
**Upper Lip Angle**					
T0	Mean ± sd	5.1± 4.9	4.8 ± 5.7	8.2 ± 7.5	0.203 ^A^
T1	Mean ± sd	5.1 ± 0.9	8.7 ± 6.0	3.8 ± 5.5^2^	0.027 *^,A^
T0/T1 Change	Mean ± sd		3.9 ± 4.5	−4.4 ± 6.6	<0.001 *^,t^
*Intra Group Change p*			0.002 *^,P^	0.014 *^,P^	
**Upper Lip Length**					
T0	Mean ± sd	18.2 ± 2.0	17.7 ± 1.3	18.0 ± 2.4	0.691 ^A^
T1	Mean ± sd	18.2 ± 2.0	18.5 ± 1.9	18.6 ± 1.6	0.837 ^A^
T0/T1 Change	Mean ± sd		0.8 ± 1.7	0.6 ± 2.4	0.750 ^t^
*Intra Group Change p*			0.062 ^P^	0.327 ^P^	
**Upper Lip Thickness**					
T0	Mean ± sd	12.9± 2.1	13.1 ± 1.7	12.6 ± 3.3	0.786 ^A^
T1	Mean ± sd	12.9 ± 2.1	13.3 ± 2.3	13.2 ± 2.6	0.889 ^A^
T0/T1 Change	Mean ± sd		0.2 ± 1.3	0.6 ± 2.9	0.551 ^t^
*Intra Group Change p*			0.625 ^P^	0.388 ^P^	
**Anterior Upper Lip–TVL**					
T0	Mean ± sd	1.4 ± 0.9	1.0 ± 1.2	1.9 ± 2.3	0.230 ^A^
T1	Mean ± sd	1.4 ± 0.9	1.5 ± 1.3	1.0 ± 1.2	0.347 ^A^
T0/T1 Change	Mean ± sd		0.5 ± 1.3	−0.9 ± 2.3	0.024 *^,t^
*Intra Group Change p*			0.106 ^P^	0.106 ^P^	
**Upper Lip–E Line**					
T0	Mean ± sd	−4.3 ± 2.5	−4.3 ± 2.9	−4.2 ± 1.9	0.991 ^A^
T1	Mean ± sd	−4.3 ± 2.5	−4.2 ± 3.8	−4.4 ± 1.7	0.979 ^A^
T0/T1 Change	Mean ± sd		0.0 ± 2.3	−0.2 ± 1.5	0.711 ^t^
*Intra Group Change p*			0.992 ^P^	0.507 ^P^	
**Lower Lip–E Line**					
T0	Mean ± sd	−2.8 ± 2.3	−3.1 ± 3.5	−1.7 ± 2.2	0.314 ^A^
T1	Mean ± sd	−2.8 ± 2.3	−2.9 ± 3.5	−2.3 ± 2.1	0.788 ^A^
T0/T1 Change	Mean ± sd		0.2 ± 1.6	−0.6 ± 1.5	0.146 ^t^
*Intra Group Change p*			0.544 ^P^	0.158 ^P^	
**A’-TVL**					
T0	Mean ± sd	−1.9 ± 0.5	−2.3 ± 0.7	−1.2 ± 0.8 ^1,2^	<0.001 *^,A^
T1	Mean ± sd	−1.9 ± 0.5	−2.2 ± 0.9	−2.1 ± 0.8	0.520 ^A^
T0/T1 Change	Mean ± sd		0.1 ± 1.0	−0.9 ± 0.9	0.006 *^,t^
*Intra Group Change p*			0.695 ^P^	0.001 *^,P^	

^A^: ANOVA test, ^t^: *t*-test, ^P^: Paired *t*-test, T0: pre-treatment, T1: post-treatment, sd: standard deviation, *p*: significance level, *: *p* < 0.05. ^1^: difference with control group, ^2^: difference with space opening group, ^3^: difference with space closure group.

## Data Availability

All data supporting the results of this study are included within the article. The data are currently not publicly available as they will be used in another study still in progress.
